# Exendin-4 Improves Blood Glucose Control in Both Young and Aging Normal Non-Diabetic Mice, Possible Contribution of Beta Cell Independent Effects

**DOI:** 10.1371/journal.pone.0020443

**Published:** 2011-05-31

**Authors:** Rongrong Fan, Zhanfang Kang, Lan He, Juliana Chan, Gang Xu

**Affiliations:** 1 Department of Medicine and Therapeutics, The Chinese University of Hong Kong, The Prince of Wales Hospital, Shatin, Hong Kong, China; 2 Hong Kong Institute of Diabetes and Obesity, Shatin, Hong Kong, China; 3 Li Ka Shing Institute of Health Sciences, The Chinese University of Hong Kong, The Prince of Wales Hospital, Shatin, Hong Kong, China; University of Bremen, Germany

## Abstract

**Aims:**

Type 2 diabetes is highly prevalent in the elderly population. Glucagon like Peptide-1 mimetic such as exendin-4 augments post-prandial insulin secretion. However, the potential influence of aging on the therapeutic effects of this peptide has not been well studied. In this study, we examined the glucose regulatory effects of exendin-4 in mice with different ages.

**Methods:**

We treated 3-month and 20 to 22-month old C57/DBA mice with 10 nM/kg exendin-4 for 10 days with measurements of blood glucose and body weight. We performed OGTT and ITT to evaluate the glucose response and insulin sensitivity. Islet morphology and beta cell mass were measured by immuno-staining and beta cell proliferation was evaluated by BrdU incorporation and PCNA staining. Real-time PCR and western blot were used to measure protein changes in the liver tissue after exendin-4 treatment.

**Results:**

Exendin-4 treatment improved glycemic control in both 3-month and 20 to 22-month old mice. In both groups of mice, the blood glucose lowering effect was independent of beta cell function as indicated by unchanged beta cell proliferation, insulin secretion or beta cell mass. Moreover, we found that exendin-4 treatment increased hepatic AKT and FOXO1 phosphorylation and inhibited glucose-6-phosphotase (G6P) and Phosphoenolpyruvate carboxykinase (PEPCK) expression in young mice, but this effect was attenuated in aging mice while the insulin sensitivity showed no change in the young group but significantly improved in aging mice.

**Conclusion:**

Based on these data, we conclude that the glucose lowering effect of exendin-4 in normal non-diabetic mice was not blunted by aging. We further showed that although there was slight difference in the glucose modulating mechanism of exendin-4 therapy in young and aged mice, the improved glucose control seemed uncorrelated with increased beta cell mass or insulin secretion.

## Introduction

Incretin based therapy has been clinically applied for the treatment of diabetes. However, the glucose regulating mechanism and potential danger are still less known and under hot discussion. In this study, we used young and aging rodent models to evaluate the potential effect of aging on Glucagon like peptide-1 (GLP-1) mimetic exendin-4 therapy.

Exendin-4 is a DPPIV resistant GLP-1 receptor agonist [1]. Exendin-4 exerts insulinotropic effects and has multiple glucose regulatory functions through activation of GLP-1 receptor in the mammalian cells [2]. Exendin-4 treatment increases proliferation, neogenesis and survival of beta cells through activation of PKA and AKT with associated gene expression [3], [4], [5], [6], [7], [8]. Treatment with exendin-4 increases satiety, reduces food intake and slows gastric emptying [9], [10], [11], [12], [13]. In adipocytes, exendin-4 enhances insulin sensitivity and glucose transport by increasing the expression of Insulin Receptor beta (IR beta), Insulin Receptor Substrate-1 (IRS-1) and Glucose Transporter 4 (GLUT4) [14], [15]. In the murine liver, exendin-4 treatment improves glucose and lipid metabolism[16], independent of insulin disposal although the exact mechanism remains to be clarified [17]. It was also reported that exendin-4 inhibited hepatocyte and cholangiocyte apoptosis [18], [19].

The risk of diabetes increases with age which is also a risk factor for drug-induced hypoglycemia. Thus, GLP-1 mimetics may be preferred in elderly subjects due to their low risk of hypoglycemia. Despite these theoretical advantages, the effects of aging on incretin therapy have not been well studied. Both beta cell function and proliferation decline with aging[20] and while the GLP-1 mediated acute insulinotropic effect of exendin-4 is maintained in adult and aged rodent, the drug has no effect on beta cell proliferation [21], [22]. With aging, there are also downregulated key signaling molecules downstream of the GLP-1 receptor, such as glucokinase, pancreatic and duodenal homeobox 1 (Pdx-1), insulin and GLUT2 expression in the pancreatic islets [23], [24]. In this study, we systematically evaluated whether the therapeutic effects of exendin-4 still maintain in aging rodent models.

Previous studies on exendin-4 action were mostly done in diabetic rodent models. Since beta cell function can be influenced by prevailing blood glucose and lipid level as well as peripheral insulin sensitivity, the blood glucose lowering effect of exendin-4 may be a non-specific effect due to amelioration of gluco- or lipo-toxicity via improvement in the peripheral tissues. In this study, we used mice with normal glucose tolerance to evaluate potential infuence of aging on the glucose lowering effects of exendin-4.

## Results

### 1. Exendin-4 improved glucose response in both young and aging mice

**Figure 1:**
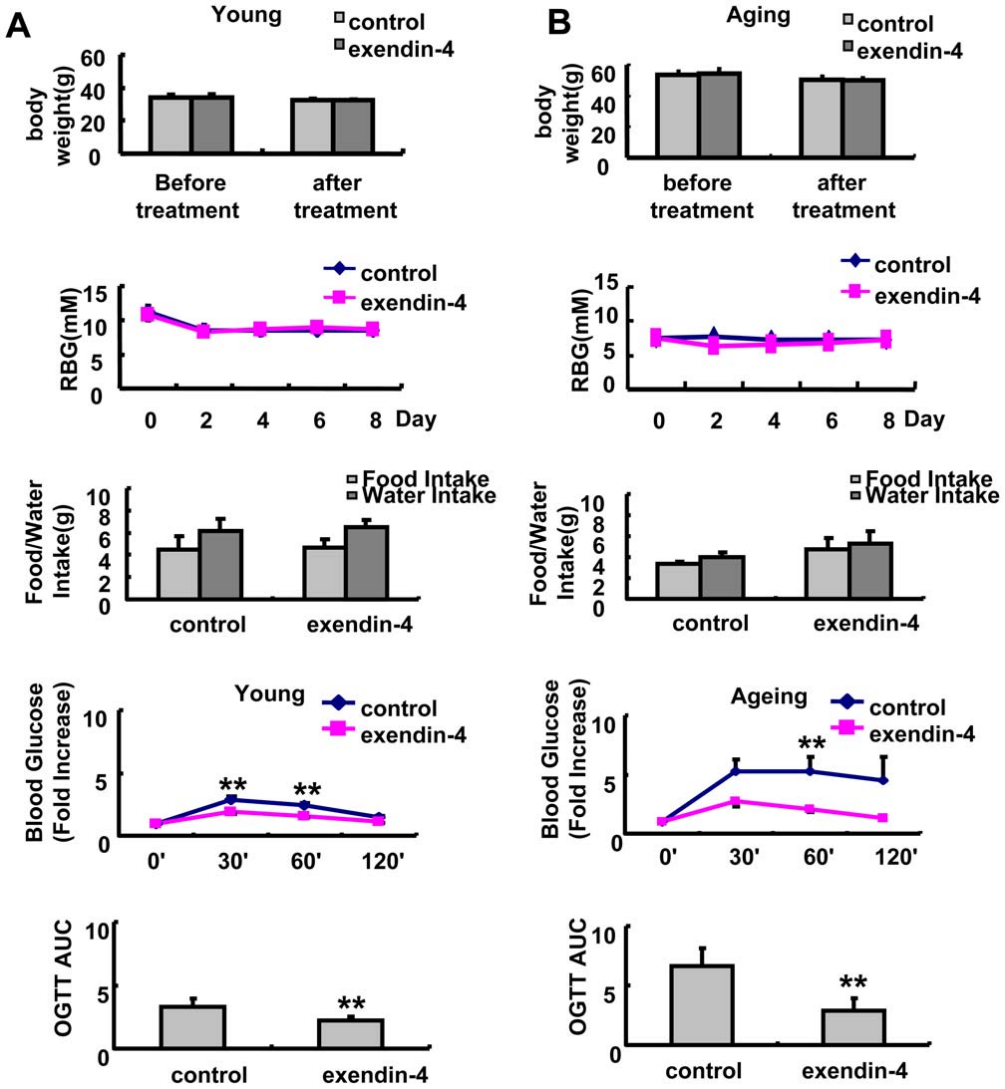
Effect of exendin-4 treatment on body weight, blood glucose, food and water intake and glucose response in A,3 months and B,20–22 months old mice, n = 7 in each group of 3 months old mice, n = 5 in each group of 20–22 months old mice. The values were shown as Mean±SEM, **P<0.05 vs control group, ***P<0.01 vs control group.

Body weight and random blood glucose were not changed after exendin-4 treatment in both groups of mice (Figure 1). However, the OGTT test showed significantly improved glucose control in both young and aging mice(Figure 1). However, the 24 hour food and water intake was not altered by exendin-4 therapy in both groups of mice, suggesting that the reduced food intake was not responsible for the improved glucose response in exendin-4 treated mice(Figure 1).

### 2. Insulin secretion was significantly lower in exendin-4 treated young and aging mice

**Figure 2:**
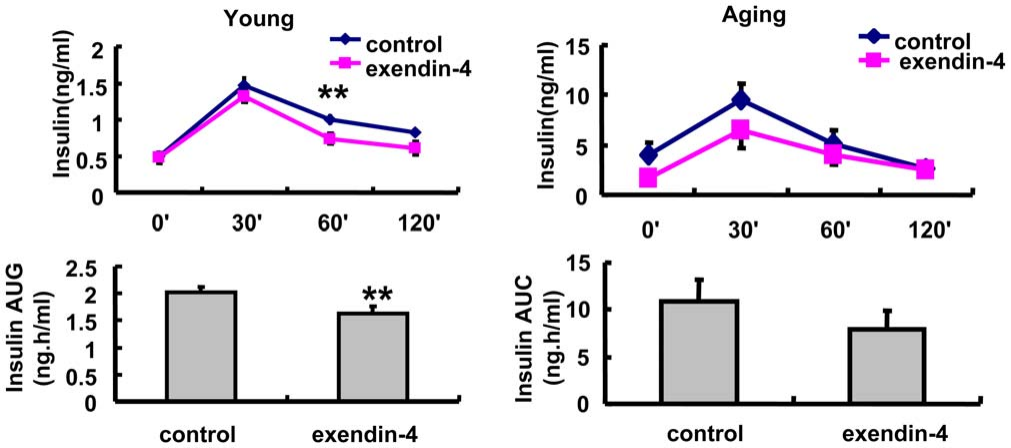
Glucose stimulated insulin secretion in OGTT time points in A, 3 months and B, 20–22months old mice treated with PBS or exendin-4, n = 7 in each group of 3 months old mice, n = 9 in each group of aging mice. Values were shown as Mean±SEM, **P<0.05 vs control as determined by student t-test.

Exendin-4 increased beta cell mass and restored insulin secretion in multiple diabetic models. Interestingly, according to our data, although the OGTT showed significant improvement in both groups of mice, the insulin levels during OGTT were decreased by exendin-4 treatment in both young and aging mice. This decline reached significance at 60 min in 3-months old mice (Figure 2A). These data suggested that in the normal non-diabetic mice, exendin-4 improved glucose control through an insulin-independent mechanism.

### 3. Beta cell mass and proliferation were not changed in either young or aging mice

**Figure 3:**
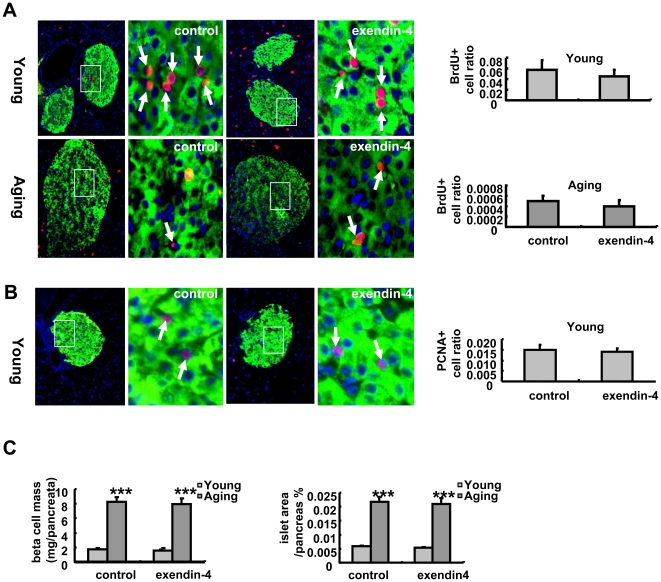
Beta cell mass and proliferation evaluation. Beta cell proliferation was evaluated by BrdU staining, the BrdU positive cells (red) to the insulin secreting cell (green) ratio was quantified (Figure 3A). PCNA staining was performed in 3 months old mice and the PCNA positive cells (red) to insulin secreting cell (green) ratio was quantified (Figure 3B). The beta cell mass and islet/pancreas % was also evaluated (Figure 3C) The values were shown as Mean±SEM.

Exendin-4 has been reported to improve beta cell regeneration and increase beta cell mass. However, in both 3-months and 20 to 22-months old mice, 10 days' treatment with exendin-4 did not significantly promote beta cell growth. The beta cell morphology was normal and the beta cell mass were not changed in both groups of mice. BrdU is a nuclear acid mimetic which is incorporated into genomic DNA during cell proliferation. We stained the pancreas with BrdU and insulin antibody and quantified the ratio of BrdU positive cells in each islet of the pancreas. In aging mice, the beta cell proliferation rate was very low and showed no difference in both saline and exendin-4 treated groups (Figure 3A). In the 3-month old mice, exendin-4 treatment did not increase beta cell proliferation either. The quantification results suggested a lower trend of proliferation rate in the islets of exendin-4 treated group (Figure 3A),. The PCNA staining results also showed no difference in 3-months old mice, in the aging group, we failed to detect any PCNA positive cells in the pancreatic islets, suggesting that the beta cell proliferation rate was very low in aging mice (Figure 3B). In consistence with this result, beta cell mass and islet area/whole pancreas ratio did not show any increase by exendin-4 treatment in both young and aging mice (Figure 3C).

### 4. Insulin sensitivity was slightly improved by exendin-4 treatment in aging but not young mice

**Figure 4:**
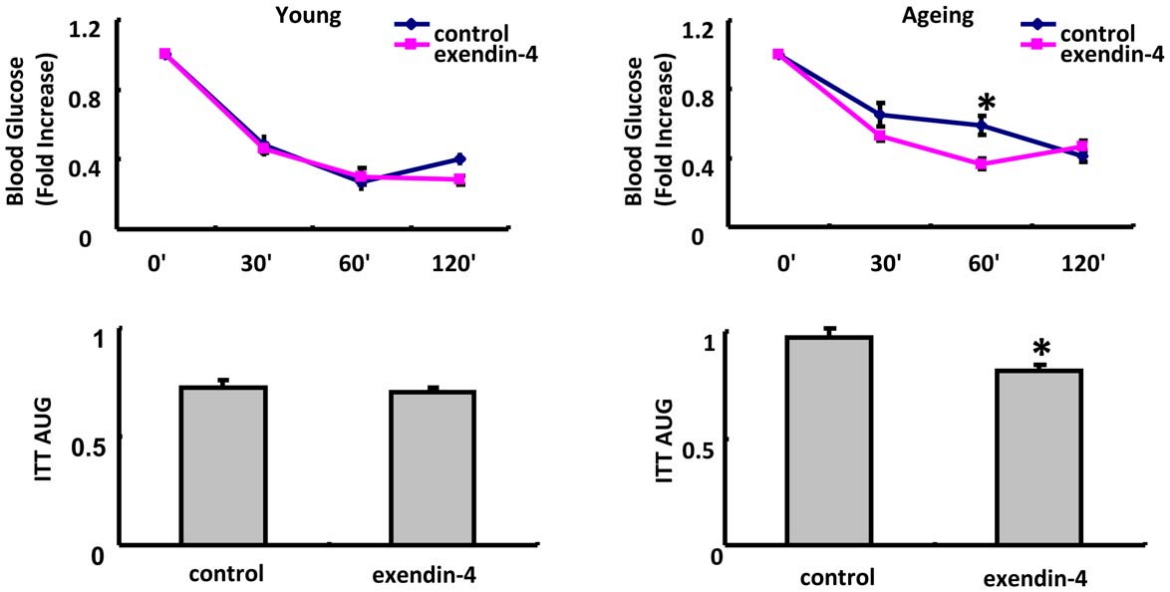
Insulin Tolerance Test(ITT) in 3 months old mice and 20–22 months old mice. N = 6 in each group of 3 months old mice and n = 4 in each 20–22 months old mice. The Values were presented as Mean±SEM, *P<0.05 as determined by student test.

ITT was performed to evaluate the insulin sensitivity in young and aging mice. After exendin-4 treatment, insulin sensitivity was significantly improved in aging group But was not significantly changed in young mice treated with exendin-4 (Figure 4).

The fasting glucose insulin and glucagon profile was evaluated in both young and aging mice(Table.1). Based on the results, we found that although blood glucose was not significantly different in all four groups of mice, the insulin level was significantly increased in aging group, suggesting enhanced insulin resistance in aging mice.

**Table 1:** Glucose, insulin and glucagon profile in young and aging mice treated with PBS or exendin-4.

	Young		Aging	
	control	exendin-4	control	exendin-4
Fasting Glucose(mM)	7.46±1.13	7.65±0.86	6.20±1.12	5.80±0.98
Insulin (ng/ml)	0.49±0.21	0.48±0.23	3.98±3.11^ab^	1.69±0.24^ab^
Glucagon(pg/ml)	144.56±19.85	191.71±33.60	236.55±4.68^ab^	215.68±39.89^ab^

n = 13 in each 3-months old group, n = 9 in each aging group. a, p<0.05 vs young control; b, p<0.05 vs young exendin-4 as determined by Oneway-ANOVA test. The values were shown as Mean±SEM.

The glucagon level was not significantly inhibited by exendin-4 in both groups of mice and in aging mice, serum glucagon level was also significantly higher comparing with young mice (Table.1).

### 5. Exendin-4 inhibited glucose-6-phosphatase (G6Pase) and phosphoenolpyruvate carboxykinase (PEPCK) expression in the hepatocytes in young adult mice but not aging mice

**Figure 5:**
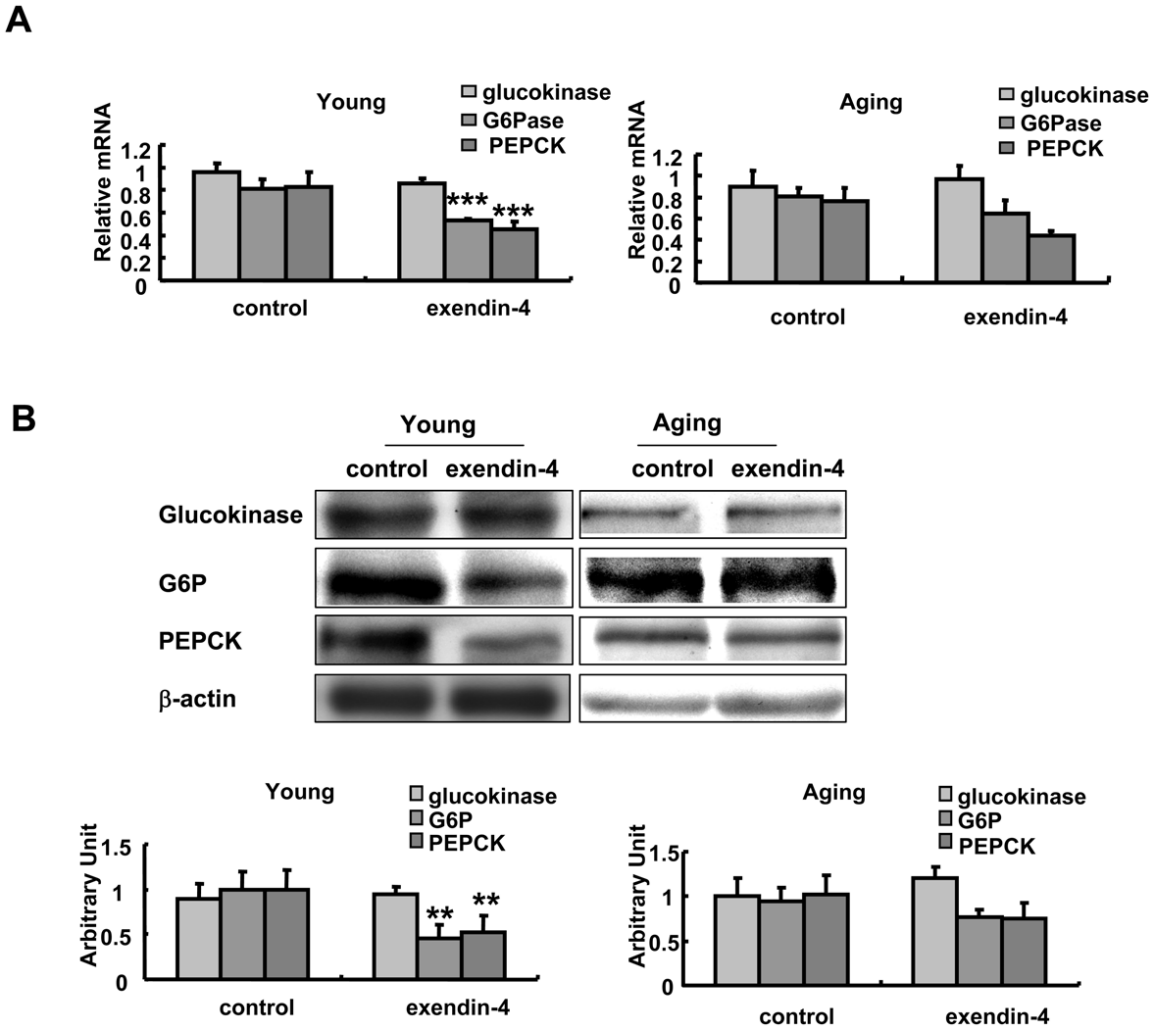
Expression of Glucokinase, G6Pase and PEPCK level in young and aging mice treated with PBS or exendin-4. n = 7 in each group of 3 months old mice, n = 5 in each group of aging mice. A, mRNA expression. B, protein level, the western blot was semiquantified. Values were shown as Mean±SEM, ***P<0.01 vs control group as determined by student's t test.

Glucose homeostasis is mainly regulated through balanced glucose output and uptake in the hepatocyte. As the improved blood glucose response in both young and aging mice were not accompanied by increased insulin secretion or beta cell mass, we further checked the glucose metabolism in the liver by examining the expression level of a series of genes participating in hepatic gluconeogenesis and glucolysis in the exendin-4 treated mice. In both 3 and 20 to 22-months old mice, glucokinase expression was not changed by exendin-4. However, exendin-4 therapy inhibited both G6Pase and PEPCK in 3-months old mice but in aging mice, this effect was attenuated (Figure 5). The western blot results were consistent with the real-time PCR data. These results suggested that inhibited liver gluconeogenesis was at least partially involved in the glucose lowering effect of exendin-4 in 3-months old but was attenuated in aging mice.

### 6. AKT but not AMPK phosphorylation was activated by exendin-4 in young but not aging mice

**Figure 6:**
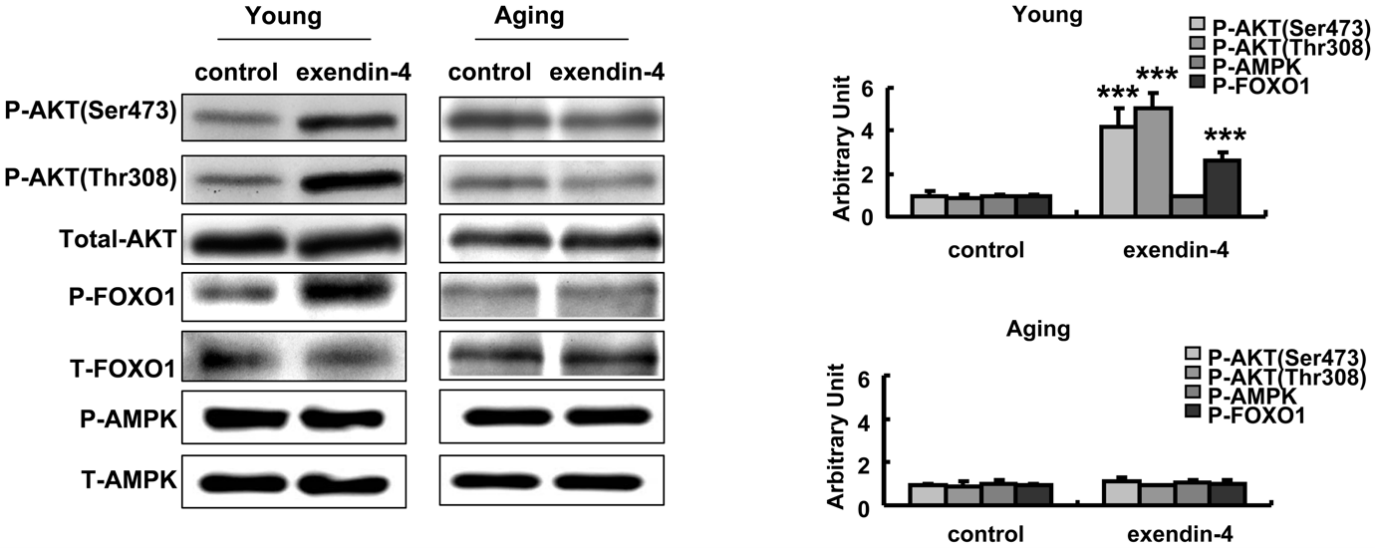
Exendin-4 induced AKT and FOXO1 phosphorylation in young mice but not aging mice. Phospho-AKT, FOXO1 and AMPK were detected in liver tissues of both young and old mice, the western results were semiquantified (Fig.5). Values were shown as Mean±SD, ***P<0.01.

Both AMPK and AKT are key kinases which regulate glucose metabolism in the hepatocytes. In our system, liver AMPK phosphorylation was not altered by exendin-4 treatment. In parallel with the G6P and PEPCK changes, AKT phosphorylation level at both Ser473 and Thr308 sites were increased in exendin-4 treated young adult mice but not aging mice (Figure 6A). The increased AKT phosphorylation was paralleled with enhanced FOXO1 phosphorylation, a well established regulator of liver gluconeogenesis (Figure 6A). The enhanced AKT phosphorylation was not accompanied by upregulated insulin level with similar fasting insulin levels in both groups of mice.

## Discussion

In this study, we examined the effect of aging on therapeutic responses of exendin-4 in 3-month and 20 to 22-month old C57/DBA mice with normal glucose tolerance to examine whether aging impaired exendin-4 therapy. After 10 days' exendin-4 treatment, both groups of mice showed significant improvement in glucose control. Increased insulin secretion might not be involved in the glucose-regulatory effect of exendin-4 in our normal non-diabetic model as insulin secretion level was not significantly increased in both groups of mice after exendin-4 treatment. The current data was consistent with our previous published results, although exendin-4 treatment significantly increased beta cell regeneration and improved glucose control in pancreatectomized rats, the insulin level was actually substantially but not significantly decreased by exendin-4 treatment [3]. This suggested that the non-insulin effects of exendin-4 may also play a major glucose modulating role. In both young and aging groups, the islet area and beta cell mass were not altered by exendin-4 treatment. In consistence, according to the in vivo BrdU incorporation assay and PCNA staining results, the rate of beta cell proliferation was very low in aging mice both in the PBS and exendin-4 treated groups, while in the 3-months old mice, beta cell proliferation rate was slightly but not significantly reduced after exendin-4 treatment. This might be due to improved glucose response after exendin-4 treatment. In addition, in both young and aging mice, we did not observe significant increase in AKT phosphorylation after exendin-4 treatment (Figure S2).

Previous studies in young diabetic animal models suggested exendin-4 augmented beta cell function. However, as beta cell proliferation and insulin secretion were highly correlated with circulation glucose and lipid, it is therefore difficult to identify the non-beta cell effects due to improvement in glucose and lipid profile. Also, it is not easy to clarify whether the increased beta cell mass, which have been found in multiple diabetic rodent models after exendin-4 treatment, was due to increased beta cell proliferation or decreased beta cell apoptosis or both. In contrast to the diabetic mice models, the non-diabetic mice showed normal glycemic control and islet morphology with very rare TUNEL positive cells in the panreatic islets (data not shown). In addition, as beta cell proliferation rate is very low, it was reasonable that 10 days' treatment was not enough to trigger obvious islet expansion in normal adult mice. In support of this, we only found significant increase in beta cell mass expansion 4 weeks after the exendin-4 intervention in the pancreatectomized rat model[3]. Indeed, in 2 months old mice, 21 days' treatment with both 10 nM and 24 nM exendin-4 significantly increased beta cell proliferation(Figure S1), but 10 days' injection with both dosages showed no obvious effect(data not shown),which suggested that exendin-4 only triggered beta cell proliferation after relatively chronic treatment in young mice. Therefore, 10 nM exendin-4 treatment for 10 days in both young and aging mice was appropriate to study the extra-pancreas effects of GLP-1 mimetics. This result is consistent with previously published literature. [20].

Recently, the blood glucose lowering effects of exendin-4 independent of beta cells have attracted much attention. In *in vitro* experiments, exendin-4 enhanced insulin signaling pathway although it remains to be confirmed in *in vivo* conditions[25]. In our experiment, the ITT results showed significantly improved insulin sensitivity in aging mice but not in the 3-months old young mice after exendin-4 treatment. This result was reasonable because the insulin sensitivity in 3-months group was already very ideal comparing to the aging mice. To explain how exendin-4 lowered glucose in those young adult mice, we checked the liver glucose metabolism after exendin-4 treatment in 3 months old mice. Liver is a major organ in maintaining glucose homeostasis by balancing gluconeogenesis, glycogenolysis and glycogen synthesis. In exendin-4 treated 3-month old mice, the liver G6Pase and PEPCK expression level were decreased while that of glucokinase was not altered. These results are consistent with inhibition of hepatic gluconeogenesis with reduced glucose output. The changes were accompanied by increased AKT activity, as evidenced by elevation of ser473 and thr308 phosphorylation of AKT as well as FOXO1 phosphorylation, but not AMPK. Since FOXO1 positively regulates G6Pase and PEPCK transcription [26], [27], de-activation of FOXO1 by AKT may contribute to the inhibition of gluconeogenesis by exendin-4 in these young mice. These findings were not observed in the aging group. Previously published findings from young or adult obesity models showed that adenovirus mediated transduction of GLP-1 reduced liver glucose output by inhibiting PEPCK and G6Pase expression in obese mice [16], [28], [29], which is consistent with our current findings in young mice. However, no studies were performed to evaluate the therapeutic role of exendin-4 in aging obesity models. Comparing with young mice, our aging mice were highly obese and insulin resistant, which was close to young obesity rodent models in phenotype. It is surprising that the liver response to exendin-4 seemed to be significantly blunted. The therapeutic effects of exendin-4 in the liver of ageing mice need to be further explored.

In line withn our findings, *in vitro* activation of GLP-1R also causes AKT phosphorylation in hepatocytes [28] although the detailed mechanism is not fully elucidated. In the beta cells, GLP-1R activation induces CREB phosphorylation with upregulation of IRS protein expression and increased AKT activity [30]. However, in our study, we did not find changes in CREB-IRS signaling in the liver after exendin-4 treatment (data not shown). Therefore based on our data, we can not get the conclusion that the effect of exendin-4 in the rodent liver was through the GLP-1 receptor. It has been suggested that GLP-1 receptor is uniquely expressed in the portal vein of the liver organ and that hepatic glucose and lipid metabolism may be regulated through a putative glucose/GLP-1 sensor in the porto-hepatic circulation [31]. Other researcher proposed expression of GLP-1R in both human hepatocyte tissue and cell lines in the regulation of incretin effects in the liver [28]. However, the existence of GLP-1 receptor in rodent liver is still very controversial. According to our data, the insulin secretion level was not significantly altered by exendin-4 treatment, and as the animals were sacrificed at least 12 hours after exendin-4 treatment, the insulinotrophic effect of exendin-4 could be excluded. Therefore, the increased AKT/FOXO1 phosphorylation was less likely mediated by insulin. One explanation for the in vivo effects of exendin-4 on the liver is the inhibited glucagon secretion [32]. Therefore, we tested the serum glucagon level in four groups of mice, the results showed that although the serum glucagon level was significantly increased by aging, it was not inhibited by exendin-4 treatment in both young and aging mice. In addition, liver AMPK, which has been reported to be phosphorylated by serum glucagon, was not altered by exendin-4 treatment in both groups of mice[33].These data suggested that glucagon might not participate in the glucose modulating effect of exendin-4 in our models. Another potential explanation is increased somatostatin. Somatostatin secretion has been reported to be stimulated by exendin-4[34], [35], [36]. The glucose modulating mechanism of somatostatin based therapy was contraversial, several previous studies showed that somatostatin normalized blood glucose potentially by inhibiting glucagon secretion[37], [38], [39]. However in our system, the glucagon level was not significantly altered. Therefore increased somatostatin was less likely to participate in the improved glucose response in our model. But we currently do not have ELISA data to exclude this possibility.

Since incretin receptor expression level is tightly regulated by prevailing blood glucose level at least in the pancreatic islets of human and rodents [40], it remains plausible that the inhibitory effect of incretin therapy on hepatic gluconeogenesis might be due to changes in other receptors or pathways yet to be identified.

Other researchers have shown that GLP-1 increased beta cell proliferation by regulating IGF-1 receptor expression in the beta cells [41]. Herein, interruption of IGF-1 receptor significantly abrogated the proliferative and anti-apoptotic effects of GLP-1. As AKT is one of the major kinases involved in IGF-1 receptor signaling pathway, we also examined the expression of IGF-1 receptor expression in both groups of mice which remained unchanged by exendin-4 treatment in both young and old mice (data not shown). Taken together, we hypothesize that the increased AKT phosphorylation in the liver might be mediated through a signaling pathway different from that in the beta cells.

In aged rodent models with dysglycemia, GLP-1 infusion augments insulin secretion and improves glycemic control [42], [43]. However, beta cell proliferation capability was attenuated with aging [9], [21] and treatment with exendin-4 only caused minimal mitosis in pancreatic islets of aged normal adult mice [21]. Moreover, latest evidence showed very rarely observed beta cell proliferation and neogenesis in the human islets [44]. The age-related phenomena might be due to downregulation of key transcription factors and kinases implicated in beta cell survival and mitosis [21], [24], [45]. In keeping with these findings, we also observed very slow rate of beta cell proliferation in the aging mice. Therefore, our findings and others suggest that the therapeutic effects of exendin-4 mediated through beta cell regeneration might not be clinically important in human adults. These evidence raised the concern that the effect of incretin based therapy on the beta cells might be affected by aging.

Nevertheless, based on our data, we demonstrated that the glucose-lowering effects of exendin-4 were preserved in aging non-diabetic mice. These data strongly supported that although exendin-4 mediated beta cell improvement might be affected by aging, the non-beta cell derived glucose lowering effects of exendin-4 therapy was still preserved, which maintained the therapeutic response of exendin-4 in aging diabetic patients. Exendin-4 reduced hepatic gluconeogenesis in young mice and enhanced insulin sensitivity in old mice, both resulted in normalized blood glucose. The lack of effects of exendin 4 on GLP1 and IGF1 receptors also suggest that other novel pathways may participate in the exendin-4 effect on the liver. These results offer new insights into the age-related changes in glucose metabolism which has bearing on the use of incretin-based therapy in elderly population.

## Methods

### Animals and treatment

C57BL/DBA mice were maintained in a 12-h light/dark cycle with free access to diet and water. Both 3-month and 20 to 22-month old mice were treated with 10 nM/kg exendin-4 in Phosphate buffered saline(PBS) or PBS alone by intraperitoneal injection in the morning once daily for 10 days. 1 g/L BrdU was added into the drinking water during the exendin-4 or PBS treatment. Body weight and random blood glucose were measured every two days. Oral glucose tolerance test (OGTT) was applied on the 10^th^ day before exendin-4 treatment. After the OGTT test, the mice will continue to receive exendin-4 or PBS injection on the 11^th^ day and sacrificed for tissue analysis on the 12^th^ day. All procedures were in accordance with the guidelines for care and use of laboratory animals and approved by The Animal Subjects Committee of the Chinese University of Hong Kong.

### Oral Glucose Tolerance Test (OGTT)

After 9 days of treatment, the mice were fasted for 12 hours before OGTT. Each mouse was gavaged with 3 g/kg glucose. Blood glucose was measured at 0, 30 60 and 120 min after OGTT using One-touch Glucose Ultrameter (Johnson-Johnson). The area under the curve (AUC) was calculated using the formula AUC = 0.25×G0+0.5×G1+0.75×G60+0.5×G120 where G0, G30, G60, G120 were blood glucose level at each time point during OGTT.

### Insulin enzyme-linked immunosorbant assay (ELISA)

Fasting insulin and insulin secretion at each OGTT time point were measured. Blood samples (about 100 μl) were collected from the tail tips. This was achieved by cutting the tail tip of each mouse with a scalpel blade followed by gently stripping of blood from the tail tips into a heparinized capillary tube. The blood samples were centrifuged at 4000 rpm at 4°C for 15 minutes and the supernatant plasma was collected for insulin analysis. Plasma insulin was measured using the rat/mouse insulin ELISA kit (Millipore) according to the manufacturer's instructions. Insulin AUC was calculated by the formula AUC = 0.25×I0+0.5×I30+0.75×I60+0.5×I120 where I0, I30, I60, I120 were insulin levels at each time point during OGTT.

### Histological staining

Pancreatic islet morphology and BrdU incorporation were quantified after treatment with exendin-4. Briefly, formalin fixed and paraffin-embedded 4μm sections of pancreas were deparifinized and dehydrogenated. After antigen retrieval, the sample slides were stained with guinea-pig anti-insulin, rat anti-PCNA (DAKO, 1∶100), mouse anti-glucagon and rat anti-BrdU (Accurate Chemical & Scientific, 1∶200) overnight at 4°C, followed by staining with cy2-goat anti-guinea pig, cy3-donkey anti mouse or cy3-donkey anti-rat antibody (Jackson, 1∶400) at room temperature for 2 hours. The sample slides were washed three times with 0.1% TBST and stained with DAPI (invitrogen) before microscopic analysis. Insulin positive and BrdU positive cells in each islet were quantified and BrdU+/Insulin+ ratio was determined to evaluate beta cell proliferation in the pancreatic islets. The islet and beta cell mass were determined as previously described[3]. Briefly, two pancreatic sections of each block were stained for each animal. Insulin positive area vs total pancreas area was evaluated using imageJ. The beta cell mass was quantified by multiplying the pancreas weight with the insulin positive area.

### Insulin Tolerance Test(ITT)

Insulin sensitivity was evaluated by ITT test after 9 days' exendin-4 treatment. Briefly, all the mice were starved for 5 hours before ITT. 1 IU/kg insulin was injected by ip and blood glucose at 0′, 30′, 60′ and 120′ was tested. AUC was calculated using the formula AUC = 0.25×I0+0.5×I1+0.75×I60+0.5×I120. I0, I30, I60 and I120 represent the blood glucose at indicated time points.

### Real-time PCR

Total RNA was extracted using the TRIZOL kit (invitrogen) according to the manufacturer's instructions. For real-time quantification, the first strand cDNA was prepared using the Reverse Transcription Kit (Invitrogen). The primers for real-time PCR were attached in the Table S1. Real-time PCR was performed using the SYBR-Green Kit (ABI) on an ABI Prism 7000 and the expression results was normalized using beta actin as internal control.

### Western Blot

Proteins of the animal liver were analyzed by Western Blot. The protein was separated in the SDS gel and transferred to the PVDF membrane, which was further blotted with Ser473 and Thr305 Phospho-AKT, total AKT, Phospho-FOXO1 and total FOXO1 (Cell Signaling), Phospho-AMPK and total AMPK (Cell Signaling), G6Pase (Santa Cruz), PEPCK and Glucokinase (Santa Cruz). The density was analyzed by Image J and semi-quantified results were presented.

### Statistic Analysis

All data analysis was performed using SPSS 8.0, Comparisons between control group and exendin-4 treated group were evaluated by the Student's t Test. All data was presented as mean ± SEM. A P value <0.05 was considered to be significant.

## Supporting Information

Figure S1 **21 days' treatment of exendin-4 increased beta cell proliferation.** 2 months old mice were treated with 10 nM or 24 nM exendin-4 for 21 days. The beta cell proliferation rate was measured using insulin(green) and BrdU(red) immuno-fluorescent staining (A), the beta cell mass was measured(B) and islet area was evaluated(C). n = 3 in each group. **P<0.01 vs control, ***P<0.001 vs control.
(TIF)

Figure S2 **Double immuno-fluorescent staining for insulin and AKT phosphorylation(Ser473) in the pancreatic islets.** The pancreas of control and exendin-4 treated groups were double stained with insulin(green) and P-AKT(red).
(TIF)

Table S1 **Real-Time PCR primer sequences.**
(DOC)
