# Supplementary figures and images for: Exendin-4 Improves Blood Glucose Control in Both Young and Aging Normal Non-Diabetic Mice, Possible Contribution of Beta Cell Independent Effects
Source: PLoS One. 2011 May 31;6(5):e20443. doi: 10.1371/journal.pone.0020443 (PMC3105063; doi:10.1371/journal.pone.0020443)

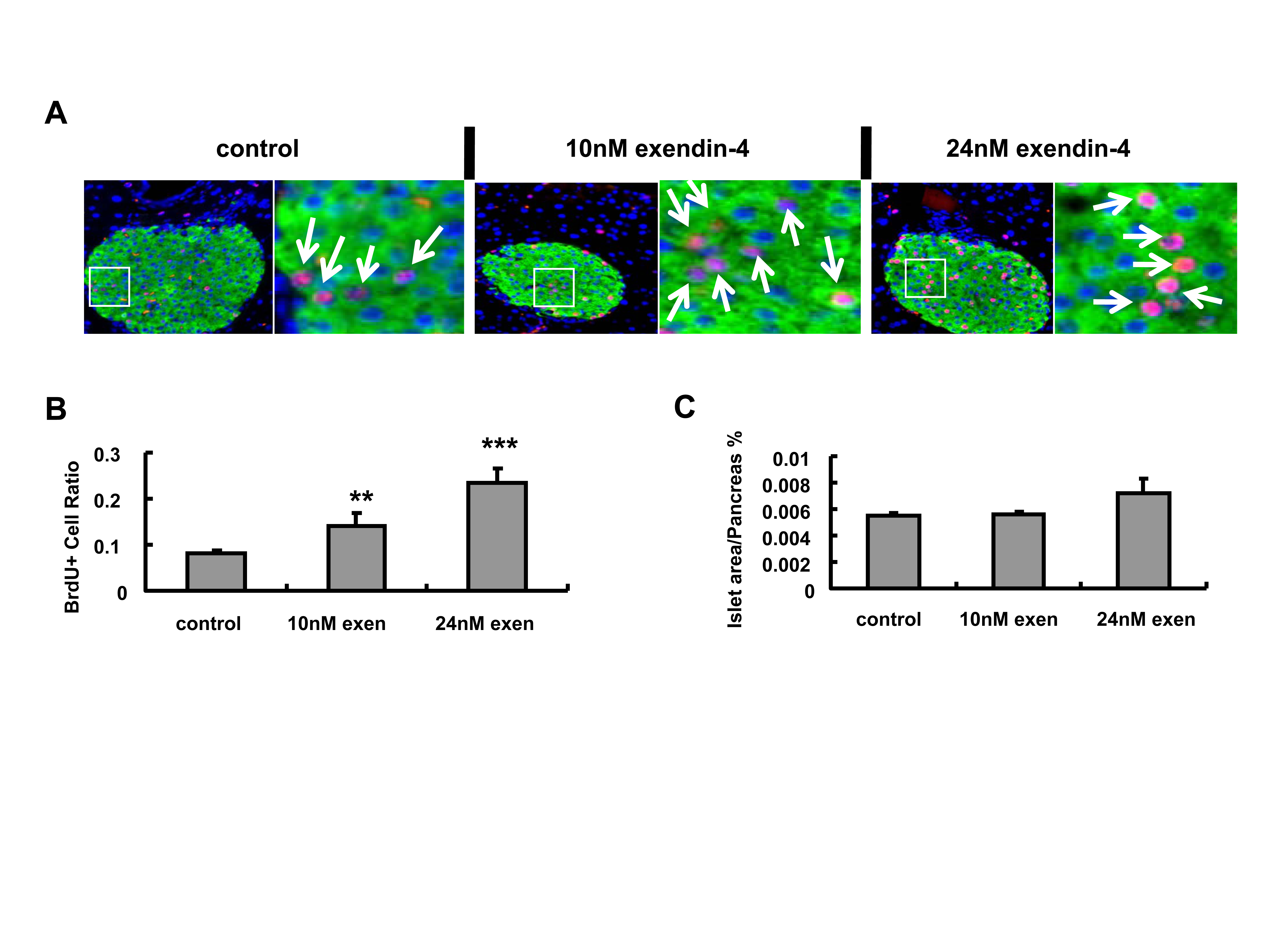

Supplement: Figure S1 — 21 days' treatment of exendin-4 increased beta cell proliferation. 2 months old mice were treated with 10 nM or 24 nM exendin-4 for 21 days. The beta cell proliferation rate was measured using insulin(green) and BrdU(red) immuno-fluorescent staining (A), the beta cell mass was measured(B) and islet area was evaluated(C). n = 3 in each group. **P<0.01 vs control, ***P<0.001 vs control. (TIF) [file pone.0020443.s001.tif]

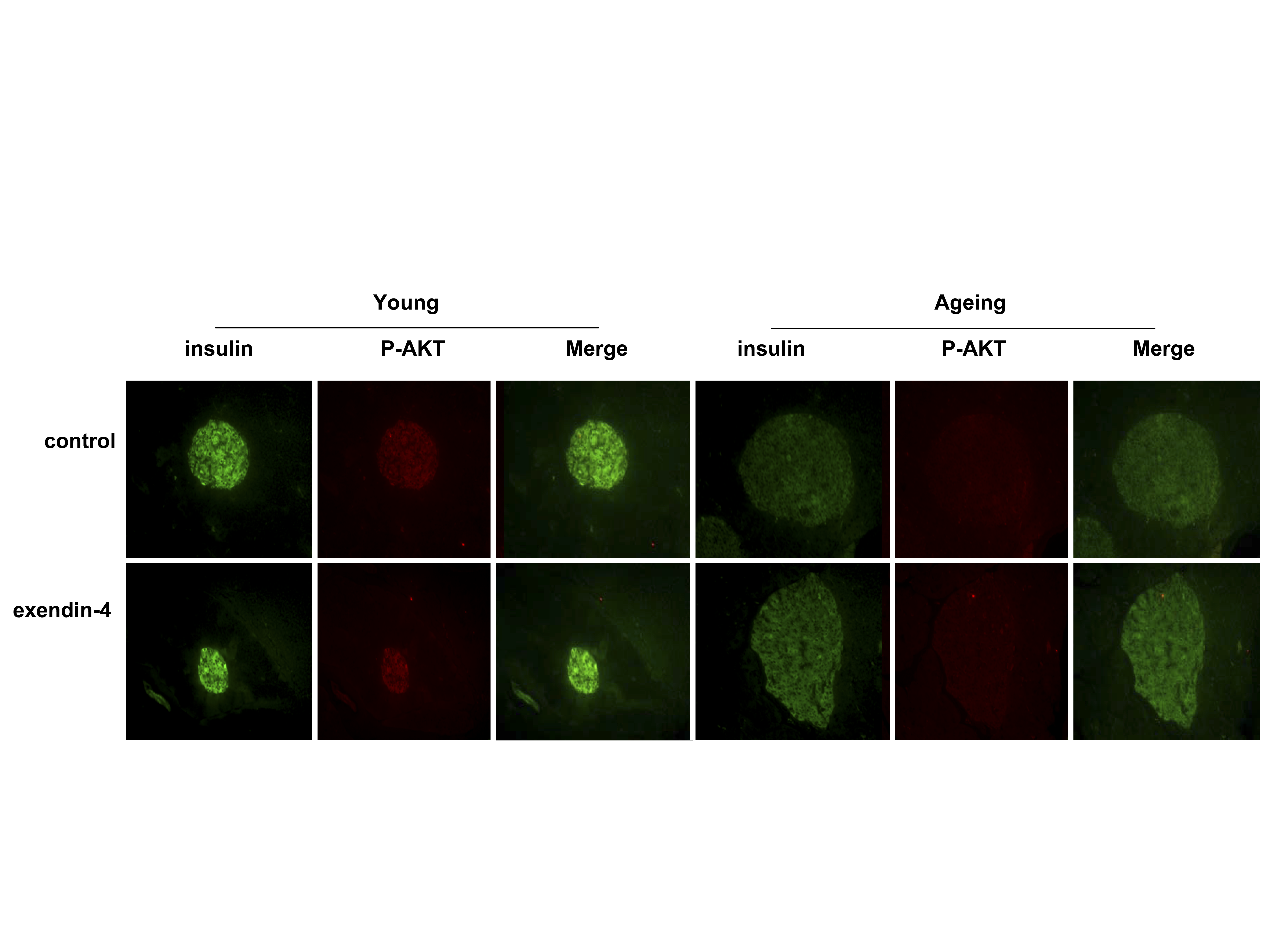

Supplement: Figure S2 — Double immuno-fluorescent staining for insulin and AKT phosphorylation(Ser473) in the pancreatic islets. The pancreas of control and exendin-4 treated groups were double stained with insulin(green) and P-AKT(red). (TIF) [file pone.0020443.s002.tif]
