# Supplementary material for: Exendin-4 Improves Blood Glucose Control in Both Young and Aging Normal Non-Diabetic Mice, Possible Contribution of Beta Cell Independent Effects
Source: PLoS One. 2011 May 31;6(5):e20443. doi: 10.1371/journal.pone.0020443 (PMC3105063; doi:10.1371/journal.pone.0020443)
Supplement: Table S1 — Real-Time PCR primer sequences. (DOC) [file pone.0020443.s003.doc]

Table S1

|  | **Forward** | **Reverse** |
| --- | --- | --- |
| **glucokinase** | **5'-CAAGCTGCACCCGAGCTT-3'** | **5'-ACTGGCCCAGCATGCAAG-3'** |
| **G6Pase** | **5'-TCCTGGGACAGACACACAAG-3'** | **5'-CAACTTTAATATACGCTATTGG-3'** |
| **PEPCK** | **5'-CTTCTCTGCCAAGGTCATCC-3'** | **5'-TTTTGGGGATGGGCAC-3'** |
| **beta actin** | **5'-TGTTACCAACTGGGACGACA-3'** | **5'-CTTTTCACGGTTGGCCTTAG-3'** |

Table.1:Real-Time PCR primer sequences.
